# Prof. R. B. Maury, M.D.

**Published:** 1872-07

**Authors:** 


Prof. R. B. Maury, M.D.—We take pleasure in adding to our list of regular contributors the name of R. B. Maury, M.D., Professor of the Principles and Practice of Medicine in the Memphis Medical College, Memphis, Tennessee. We learn that he is a most accomplished gentleman in his profession, and we hope to receive frequent contributions from his pen.

